# Socio-cultural challenges to sexual health education for female adolescents in Iran

**Published:** 2013-02

**Authors:** Robab Latifnejad Roudsari, Mojgan Javadnoori, Marzieh Hasanpour, Seyyed Mohammad Mehdi Hazavehei, Ali Taghipour

**Affiliations:** 1 *Research Center for Patient Safety and Health Quality, Department of Midwifery, School of Nursing and Midwifery,Mashhad University of Medical Sciences, Mashhad, Iran.*; 2 *Nursing and Midwifery Care Research Centre, School of Nursing and Midwifery, Isfahan University of Medical Sciences, Isfahan, Iran*; 3 * School of Nursing and Midwifery, Mashhad University of Medical Sciences, Mashhad, Iran.*; 4 *Research Center for Health Sciences, Department of Public Health, Hamadan University of Medical Sciences, Hamadan, Iran.*; 5 *Health Sciences Research Center, Department of Biostatistics and Epidemiology, School of Health, Mashhad University of Medical Sciences, Mashhad, Iran.*

**Keywords:** *Female adolescents*, *Sex education*, *Qualitative research*

## Abstract

**Background:** Despite clear reasons for necessity of sexual health education for adolescents, it is a contested issue and has faced challenges in most cultures. Providing sexual education for non-married adolescents is culturally unacceptable in most Muslim societies.

**Objective:** This qualitative study addressed socio-cultural challenges to sexual health education for female adolescents in Iran.

**Materials and Methods:** Qualitative data from female adolescents (14-18 yr), mothers, teachers, authorities in health and education organizations, health care providers and clergies were collected in two large cities of Iran including Mashhad and Ahvaz through focus group discussions and individual in-depth interviews. Data were analyzed using conventional qualitative content analysis with MAXqda software.

**Results:** Our results revealed that the main socio-cultural challenges to sexual health education for adolescents in Iran are affected by taboos surrounding sexuality. The emergent categories were: denial of premarital sex, social concern about negative impacts of sexual education, perceived stigma and embarrassment, reluctance to discuss sexual issues in public, sexual discussion as a socio-cultural taboo, lack of advocacy and legal support, intergenerational gap, religious uncertainties, and imitating non-Islamic patterns of education.

**Conclusion:** It seems that cultural resistances are more important than religious prohibitions, and affect more the nature and content of sexual health education. However, despite existence of salient socio-cultural doubtful issues about sexual health education for adolescents, the emerging challenges are manageable to some extent. It is hoped that the acceptability of sexual health education for adolescents could be promoted through overcoming the cultural taboos and barriers as major obstacles.

## Introduction

As a human right, sexual health education (SHE) has a critical role in development, promotion of equity and achievement of the millennium development goals including gender equality, reducing maternal mortality, achieving universal access to reproductive health and combating HIV/AIDS (1, 2). 

Although both adults and adolescents themselves across the world prefer that adolescents not to be engaged in sexuality, but global decrease in the puberty age, as well as later marriage, may result in earlier sexual initiation before marriage. Without preparation for their sexual lives, adolescents are vulnerable to serious threats such as sexual abuse, coercion, HIV and other sexually transmitted infections, pregnancy and unsafe abortion. It is notable that all of these threats are preventable (2, 3). There is evidence that equipping adolescents with age- appropriate and accurate sexual education, improves their sexual health (4). 

Despite clear reasons for necessity of SHE for adolescents, it is a contested issue and faces challenges in most cultures. There are several evidence from Africa and Asia showing cultural resistance to adolescents’ sexual education (5-9). Even in more liberal cultures, discussing sexuality for adolescents has not been without challenges; at least in the family level, parents-adolescents sexual communication has faced some difficulties (10, 11). Common concerns are associated with providing SHE for adolescents; for example many parents, teachers and policy-makers believe that it can result in early sexual activity and privation of childhood innocence. They think that it is contrary to their culture or religion (2). In policy-making and curriculum designing processes, there are some controversies about appropriate age and contents that should be taught (12). 

As gatekeepers of sexual health information for adolescents, adults define content of information that adolescents receive, whereas there is a gap between what they perceive that adolescents need and what adolescents themselves really need (6). Sexual education is a form of value-based education and due to political, cultural, religious and ethnical diversities, agreement on values especially in controversial areas of sexuality remains challenging (13). Asian cultures share disapproval of non-marital sex and taboos surrounding sexuality (14, 15). This is the case in Muslim countries particularly in relation to girls, because their chastity is denoting their families’ honor (16-18). In Iran and most other Muslim countries, denial of non-marital sex is an important barrier to combating HIV/AIDS. SHE programs are limited, or are skipped over by the teachers. Because of addressing many reproductive health issues in accordance with Islamic values, Iran is a successful model for other Muslim countries in some issues, but not regarding SHE for adolescents (19).

In recent years, Iranian society has experienced some degrees of modernity and Westernization that itself has influenced social traditions. Commonality of cross-gender friendship and pre- marital sex among young people is one of the most obvious changes. Access to communication technology and global media plays an important role in cultural changes. Many individuals have access to satellite television. Despite prohibition of sex outside marriage by religion and tradition, evidence shows that it has been growing in Iran especially in young people (20, 21). 

Observance of modesty, and gender segregation as strong social norms, primacy of the family and criticism of non-marital sex, all resulted in controversial perspectives about providing SHE to unmarried persons (22). 

A few studies have examined social attitudes towards sex education for adolescents in Iran, with contradictory findings. Iranian parents’ attitude towards SHE for teenagers is not positive but there is some contrary evidence indicating that both adolescents and parents agree with it (23, 24). Educating sexual health through websites seems difficult in Iran, especially in Persian language, and those programs targeting adolescents are scarce (25). 

We were interested to conduct this study due to our experiences with adolescent girls’ sexual issues such as lack of sufficient knowledge, premarital sexual activity and sexual abuse lead to pregnancy, unsafe abortion, lose of virginity and consequently stigmatization, ruining of future, being rejected from their families and running away. This is the first study aimed to explore socio-cultural challenges to sexual health education for female adolescents in Iran using qualitative approach. It is hoped that findings have some implications for policy making, designing and implementation of SHE programs and services for adolescents in Iran and other countries with the similar socio-cultural contexts. 

## Materials and methods

Qualitative approach was used for the purpose of enjoying its strengths in understanding the socio-cultural context and reflecting the voices of participants (26). Qualitative content analysis is a research method for analyzing content of text data and interpreting meaning from it (27). Study was conducted in two large province capitals in Iran, Mashhad and Ahvaz, as a religious and industrial city respectively. Participants were female adolescents and key adults including mothers, teachers, health care providers, local policy makers at health and education organizations in two provinces and also clergies. 

High Schools were recognized as appropriate settings for participants’ recruitment, holding sessions of focus group discussions (FGDs) and individual interviews, due to assistance of schools’ principals and teachers for recruitment of eligible adolescents and mothers. Because of the cultural sensitivity of study topic in Iran, especially for the reason of including student girls, organizational permissions were achieved after a prolonged process of convincing authorities. We decided to include late-adolescence group (14-18 years old, at high school), as they have had enough time to face sexual issues to be discussed. Inclusion criteria for adolescents were experiencing menstrual cycles, being unmarried and living with their family. Mothers should have had at least one daughter between 14 and 18. 

Teachers, health care providers and policy makers were included in case they had at least five years of experience in the relevant field. Clergies should have had relevant research or teaching in their experience. School assistants and teachers, assisted the researchers by introducing students and mothers who were interested in participation and discussion about the topic. Purposeful sampling with maximum variation was adopted. Totally eight high schools (three governmental and one private school in each city) in Mashhad and Ahvaz were included. 

For maintaining maximum variation, schools were selected from various socio-economic localities introduced by education organization. Within schools, students were included from all four grades and different majors. It means that researchers tried to obtain homogeneity within groups and heterogeneity between groups in terms of age, grade, major and socio-economic status. Sampling was continued until data saturation was achieved. From January 2010 until March 2011, seven FGDs (N=44) and 13 individual interviews with adolescent girls, and one FGD (N=5) and five individual interviews with mothers were conducted in two cities. 

Also five individual interviews with teachers (two biology teachers and three school counselors, all female), in two cities and one FGDs with health care providers (five female midwives who sometimes went to schools as guest speakers to talk about health issues as part of their duties) in Ahvaz were accomplished. Furthermore, heads of “Department of Schools Health” in Education organization (female) and heads of “Department of Schools and Youth Health” in health centers of two provinces (female physicians) were interviewed. In Mashahd, there were two Adolescent Friendly Service (AFS) established as part of a pilot study. 

In this AFS center Head of Department of HIV Prevention - a male physician- and a female counselor for high risk behaviors were interviewed. Additionally, a FGD with adolescent members was conducted. Looking for clergies in Mashhad, a number of male and female clergies were introduced to the researchers, from whom three (two male and one female) accepted to participate in this study. Semi-structured in-depth interviews initiated with a general question (e.g. How do you think about SHE for adolescent girls in Iran?) and guided by the interview schedule to encourage participants to express their own views. In FGDs, participants were engaged in discussing SHE for adolescent girls in Iran. All interviews and FGDs were conducted, recorded and transcribed verbatim by the second author (female). Focus group facilitator was female too. Each FGD and individual interview lasted between 60-90 minutes. 

**Statistical analysis**

Data collection and analysis were carried out simultaneously, following the principles of conventional qualitative content analysis by which coding and categorizing are originated directly from the text (27). By frequent reading of transcripts, immersion in the data in order to get an overall insight was achieved. Then the transcripts were reviewed word by word to identify meaning units. Through process of reduction and condensation, codes were emerged and categorized using MAXqda software (28). 

We attempted to enhance rigor and trustworthiness of study by prolonged engagement with participants in the phase of data collection, seeking negative cases and doing member check through giving some coded data to the participants to confirm the way that the researchers’ conceptualized the data. Peer debriefing as a process to enhance the credibility of qualitative analysis was also carried out. For this reason, samples of coded texts and interpretations were reviewed by the second and third authors who were experienced qualitative researchers and the consistency of judgments was confirmed. 

Ethics approval was obtained from Local Research Ethics Committee in Mashhad University of Medical Sciences. All participants became informed about the aim of the study and signed an informed consent form to participate in the study. In addition, for adolescents, parents’ consent was obtained as well. 

## Results

Analysis of the experiences and perceptions of participants demonstrated that the main socio-cultural challenges to provide SHE for adolescents in Iran are affected by taboos surrounding sexuality, which itself resulted in many challenges in policy-making realm, program designing and implementation and also sexual education in the families. These overlapped challenges are presented below in detail. 

**Denial of premarital sex in adolescents**

Denial of sex outside marriage in adolescents-either due to being unaware of real social issues or as a consequence of deliberate reticence- does not help to solve the problem and is a significant barrier to manage adolescents’ sexual-related complications properly. A health care provider described this as so:

*“We don’t like to think about some problems, or sometimes prefer to deny them rather than seeking solutions and, sexual health issues in our society are like this! (36Y, FGD).*

Both the health authorities and adolescents believed that this denial will repeat the experiences of AIDS in Iran. In the early years of AIDS epidemic, the policy makers did not consider the AIDS and particularly the risk of sexual transmission as a major threat for their Islamic country. However, the spread of AIDS epidemic was such that the media were forced to use the policy of clear expression of transmission ways and emphasis on protected sex.

**Social concern about negative impacts of sexual education to adolescents**

Most adults believed that sexual knowledge especially about sexual relationship causes distortion and premature sexual activity before marriage. They believed that in providing sexual education, this issue is the main concern of mothers and teachers. A mother said:

“*If we talk about sex, they get curious, their mind get busy, they say what’s the matter, they get sensitive and want to negotiate it with their friends and search more about it*.” (Mother, 35Y, FGD). 

One of the female clergies expressed her opposition to SHE and described it unnecessary and a harmful matter: 

*“In the past, we* *had no information about sex, but had no problem as well. Now, because of advanced information technology, the girls have access to some electronic information that can be destructive… Many things must be left behind. No one disagrees with the concept of education, but we have useful and non-useful knowledge. There is something that may not be useful to know*.” *(Female clergy, 43Y).*

Being concerned about the harmful nature of SHE, the secrecy is considered as a convenient and required solution by adults; While most of adolescents, by criticizing the adults, consider this concern as irrelevant and also consider their secrecy as a barrier in their access to required information. One of the adolescents elaborated the way that parents behave them when they try to seek information on sexual issues: 

*“When the media is going to train these issues they change the channel; or if we are going to read them in the book they first beat the book on our head and then wear it out; in my view the parents are the only ones that cause important problems in the understanding of sexual matters; it is the case.” (Adolescent, 18 Y, FGD).*

**Perceived stigma and embarrassment adhered to sexual issues**

Stigma and embarrassment was one of the main reasons to avoid sexual discourse, especially at the household level and this was the case for both adolescents and adults including the parents and teachers. But it is interesting that almost all participants emphasized mutual mother-daughter intimacy as the most important factor in facilitating sexual communication. A mother stated:

“…*sometimes, mother is embarrassed to discuss sexual issues, personally it is difficult for me, I like to tell her about the risks of sexual relationships, but how could I myself tell her to beware of this matter, I couldn’t really*”. (Mother, 37Y, FGD).

Adolescents expressed the behavior of adults as the main reason of feeling embarrassed to communicate about sex with them. They said that they behave in such a way that directly or indirectly prevents adolescents from talking about sex. 

“… *maybe there are thousands of questions in our mind that we embarrass to ask, they don’t allow us to tell them what we want and as a consequence we talk these things with our friends.*” (Adolescent, 16 Y, FGD).

**Reluctance to discuss sexual issues in public**

Some adults, but not adolescents, preferred to make SHE personal and private due to taboos adhered to sexual issues. They believed that public presentation of sex education can interfere with modesty and public chastity of society. A female clergy believed that SHE must be limited to the family environment because the mind of adolescents who have no sexual knowledge and experience became compromised by replying to the questions presented by adolescents who have already got sexual knowledge and experience, she noted: 

*“If you try to* *teach* *something* *,* *it gets formal* *gradually. We fear to lose the principle of modesty that we must be adhered to.* *In* *one* *class,* *many* *girls* *are modest* *and we do not have to shift their thought toward these matters. So we* *emphasize* *that* *this* *training should go* *to the* *family.”* (Female clergy, 43Y)

Also referring to the questions presented by the students in the classroom, a teacher stated: 

“*There are limitations for us. In a class with 33 studentsو we can’t explain the sexual issues. When a student asks me about the meaning of masturbation I don’t know how to explain the matter. I have to say that come* *alone to my* *consulting* *room,* *I would* *tell* *you* *about it“*(Female school counselor, 37Y).

**Sexual discussion as a socio-cultural taboo**

Speaking indirectly and deliberate avoidance of openness and transparent expression of sexual issues in order to protect modesty, sometimes cause misunderstanding. One of the clergies referring to explicitness of religious commandments about sexual issues stated that non-explicit expression of sexuality is a cultural and not a religious problem: 

“*Language* *of the prophet and Imams’* *were more* *expressive and clear* *in* *this issue. This expressiveness was to the extent that might be unacceptable in our culture. As far* *as I know, there isn’t any limitation and unreasonable stigma in relation to* *many* *areas* *of sexual health* *issues in religion.* (Male Clergy, 50Y)

A health care provider referred to importance of openness degree to be considered in order to both observing the public chastity and preventing misunderstanding. She commented in the absence of explicit expression of sexual issues, some of the teenagers will have an incorrect understanding of sexual discourse. She considered SHE as a double-edge sword, which means that sexual discourse will be harmful if it was too open or too closed and this is one of the complexities of SHE. She shared her memory: 

“*Once I went to a Middle school, one student asked me about AIDS. I said “AIDS would be transmitted through the people who have incorrect relationships; we must try to have a good behavior”. When I was going out from the class, a girl with tearful eyes came and said “Miss, I was very bad tempered with some of my friends, did I* *get* *AIDS? At that time I realized that how it is difficult to train sexual issue! I wondered what to say to this child. I said: “No, I mean street friendships, loving friendships; I never meant you get AIDS due to being bad-tempered!” It is really a double-edge sword, we may help some people but if we do wrongly and unconsciously we may devastate some others*.”(Midwife, 36Y, FGD).

**Lack of advocacy and legal support**

Due to the aforementioned challenges in the context of Iranian society, getting approval from social organizations and institutions is difficult and for promoting the program, the majority of energy and time must be used in convincing them to the priority of the sexual issue education. One authority said:

*“* *We* *need to* *explain everywhere and for everybody* *; we* *all* *need* *to take forward the work* *hardly and* *request others* *to help* *us. To go ahead, we spend most of our energy and time to convince the others and indeed not for implementing the educational program” *(Head of Schools Health Department, Female physician, 41Y). Due to fears of parents’ objection and lack of legal support, school principals also take conservative stance towards SHE and prefer to ban or restrict such trainings. A Health Care Provider viewed: 

*“Schools fear of parents’ objection. When I ask the principal why she doesn’t offer sexual education, she replies that the Education Organization does not allow. The principal maybe open-minded and want to speak about such issues but she fears. Because there isn’t any directive, so when some teachers want to talk about sexual issues in class, she emphasizes that “do not speak so open; I won’t be accountable to mothers.” (Midwife, 50Y, FGD).*

**Intergenerational gap**

Influence of Western culture and attractions on adolescents as a consequence of advanced communication technology, cause them to challenge traditions, become enamored of Western social freedoms and be interested in being free of religious constraints. This make deep gap between generations and loose adherence to religious do’s and don’ts by the adolescents. Since the ideological considerations have profound impact on providing educational content by adults as well as adolescents’ acceptance, so developing a model considering the perspectives of both generations, seems difficult. Romantic relationship is one of the typical evidence that almost all student girls referred to. Traditionally, friendship with opposite sex outside marriage even without sexual contact is not acceptable publicly in Iran. But nowadays, this phenomenon is happening as an undeniable fact in the community. The expressions presented in this regard demonstrate critical perspectives of adolescents toward traditional beliefs:

“*It is only in Iran that friendship with opposite sex* *is not acceptable yet; in other countries it is a normal issue; I don’t mean sexual relationship, in Iran even if a girl has just got a boyfriend, she is considered as a corrupt person,! Even if she’s done nothing and has had just a phone call to her boyfriend*” (Adolescent, 15 Y).

**Religious uncertainties**

Existence of uncertainties about the religious stance on some social phenomena like romantic relationships that SHE would be inevitably involved in, is another challenge in SHE. A clergy pointed that although there are “do’s and don’ts” about the romantic relationships in the Islamic jurisprudence, but due to different personal understanding of religious issues which are presented as Islamic jurisprudence, there is no general agreement among the religious scholars about necessity of SHE. So clergies who were interviewed presented two different views. An intellectual view, relying on some sexual issues in religious teachings and statements of Imams, believes that not only SHE has no religious prohibition but also seems religiously necessary:

*”When the knowledge of a girl about her sexual issues is poor, there is a possibility that she may be abused. So, religiously and culturally it isn’t good for us to provide SHE secretly and make it informal. When the religious scholars point to this matter, it means that you, as* *an educator, in the current millennium can educate sexual issues for adolescents*”. (Male clergy, 50Y)

Beside that, some clergies have traditional beliefs:

“…*it is not a good reason for sexual education if a teacher or a high-school girl says SHE is necessary; First of all it must be proved that whether SHE is necessary or not. Some of experts and I myself believe that it will cause harmful consequences*”. (Female Clergy, 43Y)

**Imitating** **non-Islamic** **patterns of education**

Using non-Islamic patterns for SHE to adolescents is one of the reasons cause religious and cultural resistance. According to the view of clergies and authorities, Muslims need to design native and culture-based patterns through relying on religious doctorine. Imitating Western models caused the lack of social acceptability of SHE. The perspective of a female clergy was in such a way: 

*“… Few years ago it was decided to implement SHE in the middle schools, but most of us didn’t agree, as we felt that this program has come from somewhere else and it is not according to our country policies and also religious jurisprudence. We assumed that many things would be open and so many issues that must be remained as secret matters in the family framework would become evident”* *.* *(Female clergy, 43Y). *

A health authority believed that promoting safe sex has been unsuccessful in adolescents’ sexual health problems. This is an evidence for the legitimacy of Quran teachings but some Muslims do not believe that:

*“The Western culture believes* *that because an adolescent may experience sex, so we should let them to know how to protect themselves! But we must find some ways to keep them away from sexual experiences. They have now found that sexual experiences must not be started in the early ages. After 20-30 years, we will say that Quran has emphasized the “abstinence” too, we try to match our Quran with their findings! While we must accept that our Quran is the most complete code of life”. (Head of Schools Health Department, female physician, 41Y)*.

## Discussion

This study revealed some overarching socio-cultural challenges to SHE for adolescent girls in Iran. Surprisingly, this study found some similarities among socio-cultural challenges regarding SHE in Iran and other cultures. Cultural challenges have been shown as a barrier to SHE in several studies of other cultures as well (5, 6, 8). 

In a young society like Iran, the time between puberty and marriage is at least 10 years and despite the religious and traditional ban of extra marital sex, it is being increased. Some Islamic organizations believe that making this issue hidden and not to pay attention to its consequences would double the trouble (29). In fact although some sexual behaviors are unacceptable in some cultures but it does not mean that these behaviors do not occur or they should be excluded from the scope of sex education (2). 

Most adults were concerned about the negative effects of adolescents’ sexual awareness. These findings have also been reported in some other studies from Iran and other cultures (6, 30). McDonald reports that adults who challenge SHE believe that the adolescents have not enough cognitive competence to understand the consequences of their performance and therefore, some sexual information that may direct them to immoral behaviors must not be presented to them. She also sites Fay and Gordon’s study who argued that not speaking about sexual issues is itself a kind of SHE (31). Evidence about the effects of SHE on adolescents demonstrates contradictory effects (32-33). 

Some evidence have illustrated positive effects of SHE like delayed sexual activity, safer sexual behaviors and decreased rate of teenage pregnancies and sexually transmitted diseases (2) However, there is some other evidence which shows mother-adolescent conversation about sex may increase sexual conduct in adolescents (34-36) however adolescents themselves believe that accurate early information about sex does not increase intention to have sex (37). 

Regarding embarrassment as one of the main barriers in sexual communication between adults and adolescents, according to “communication privacy management theory” described by Sandra Petronio, disclosing sensitive information makes people feel vulnerable; therefore people often refuse to talk about sex in order to self-protection. In addition to embarrassment, fear of parents’ judgment is another reason that makes adolescents to refuse discussing sexual issues with their parents. The more people trust their audiences, the more permeable borders become and the more they disclose their information (38). Therefore, SHE by parents can be facilitated by training child rearing skills with an emphasis on intimacy and trust between parents and children.

Observing the modesty is a principle in Islamic teachings, especially in the teachings of sexual norms, so that the Prophet of Islam considered it as the main character of religion. Even in the verses of Quran this principle has been considered in expressing the laws and norms related to sex. But obviously protecting the modesty should not resulted in lack of knowledge. 

However, some religious scholars referring to the principle of modesty infer that personalizing and privatizing the environment, content and audience**s** of SHE is one of the contributing factors in modesty development. They concluded that the principle of modesty will be destroyed if the sex education concepts became presented in public areas and will encourage adolescents to have sex (29). Considering that this inference is correct, it can be concluded that SHE is not forbidden in Islam, rather, it is conditional. This finding is important because despite numerous reports that mentioned religion as a deterrent against SHE, Islam is not against sex education but considers it as a religious requirement. 

From the perspective of Islam and Quran teachings, transcendence of human identity depends on harmonious development of different aspects of his entity including his sexuality; therefore, SHE is one of the basic necessities of human life (29).Thus, it is lack of social acceptance that hinders SHE, not religion. Recent study from Iran report parents’ negative attitude to SHE (39). So this is a very important strategy to convince parents and key adults about the necessity of SHE according to religion and lack of irrational modesty in Islam. 

As other studies have mentioned lack of legal support makes schools to hinder SHE. Even in the existence of curriculum, SHE in its implementation stage faces shortage (e.g. reducing the number of sessions) and restrictions on content (e.g. having to teach abstinence only) (40, 41). Schools give no priority to SHE and consider these programs taking the time of other courses (42).

The most important finding of this study was that cultural resistance focuses more on the nature and content of SHE programs rather than SHE itself. Most of participants, either adolescents or adults, conceived the term of “sexual education” equivalent with “education of sexual conduct”. Even, the term of SHE directed the authorities̕ and key decision makers’ thought toward “safe sex”. Due to lack of common definition for SHE and its negative meaning, it is important to choose a proper term; Because in public speaking there is no distinction among the words “Instruction, Education and Training”.

According to the Halstead and Reiss (13), SHE like any other educational course, inevitably, is a valuable activity. The purpose of SHE is more than giving knowledge about the biology of sex; it includes encouraging skills, attitudes and specific behaviors and critical reactions. Therefore policy makers and health program planners should be convinced that observance of sexual norms is almost a part of SHE program; thus the public resistance against it will be reduced. 

In other studies, intergenerational gap has been also considered as a challenge in SHE (9, 19, 43). The conflict of values between the trainers and trainees generations causes different attitudes towards sexual behaviors; it makes tension between traditional and modern approaches to sexuality which would affect SHE. 

Conducting some in-depth studies on the characteristics of SHE is suggested for confirming its consistency with religious values. It should be considered that the degree of transparency and openness in the expression of SHE must be to the extent that avoid misunderstanding beside modesty observing and paying attention to its social acceptance. Also, establishment of a proper language by a specialized team can be helpful. The findings of this study can also be useful to design SHE in other Muslim countries with the same socio-cultural contexts.

The main strength of this study was to adopt a qualitative methodology, which gave the opportunity to adolescents whose voices need to be heard and adults whom can someway influence the subject. Also, conducting the study in two cities with different cultural and religious contexts allowed the researchers to compare the findings. Contrary to our thought, the results were similar in two cities. This finding confirms coming into a global youth culture. This means that because of global communication technologies, youth are not limited to their cultural context, rather mostly depend on a global youth culture (44).

One of the limitations of this study was including only in-school adolescent girls in high-school grade; Conducting a study in which both genders participate, can increase comprehensiveness of the study, and probably lead to interesting results showing the diverse views of community about providing SHE for boys in comparison to girls. 

## Conclusion

Although there are important socio-cultural doubtful issues about SHE for adolescent girls in Iran, but it does not mean that SHE for adolescents would be impossible. Rather, the emerging challenges are manageable in some extent. Considering the sensitivity of sexual education in religious and cultural context of Iran and other countries with similar socio-cultural atmosphere, acceptability of sexual health education for adolescents could be promoted tactfully and through overcoming the cultural taboos and barriers as major obstacles. While many countries are seeking answers to this question that what kind of SHE do their adolescents need, we are still puzzled by whether adolescents should or should not get SHE.
